# 3-Chloro-*N*′-(4-diethyl­amino-2-hy­droxy­benzyl­idene)benzohydrazide

**DOI:** 10.1107/S1600536811001218

**Published:** 2011-01-15

**Authors:** Tian-Yi Li, Bing-Bing Li

**Affiliations:** a School of Chemical Engineering, Changchun University of Technology, Changchun 130012, People’s Republic of China

## Abstract

The asymmetric unit of the title Schiff base compound, C_18_H_20_ClN_3_O_2_, contains two independent mol­ecules. An O—H⋯N hydrogen bond contributes to the planarity of each mol­ecule: the dihedral angles between the two benzene rings are 12.8 (3) and 27.2 (3)° in the two mol­ecules. In the crystal, mol­ecules are linked through inter­molecular N—H⋯O hydrogen bonds, forming chains along the *a* axis.

## Related literature

For Schiff base compounds, see: Bessy *et al.* (2006 ▶); Podyachev *et al.* (2007 ▶); Raj & Kurup (2007 ▶); Pouralimardan *et al.* (2007 ▶); Bacchi *et al.* (2006 ▶); Dinda *et al.* (2002 ▶). For reference bond lengths, see: Allen *et al.* (1987 ▶). For the preparation of the title compound, see: Zhu (2010 ▶).
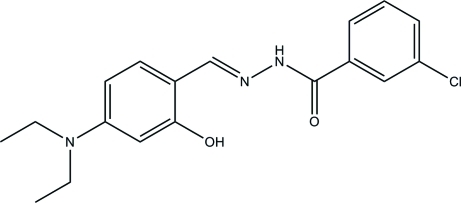


## Experimental

### 

#### Crystal data

C_18_H_20_ClN_3_O_2_
*M* *_r_* = 345.82
Triclinic, 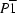

*a* = 10.087 (4) Å
*b* = 12.939 (5) Å
*c* = 14.780 (5) Å
α = 78.408 (4)°
β = 80.726 (4)°
γ = 70.632 (4)°
*V* = 1773.3 (11) Å^3^
*Z* = 4
Mo *K*α radiation
μ = 0.23 mm^−1^
*T* = 298 K
0.22 × 0.20 × 0.20 mm

#### Data collection

Bruker APEXII CCD area-detector diffractometer
Absorption correction: multi-scan (*SADABS*; Bruker, 2005 ▶) *T*_min_ = 0.951, *T*_max_ = 0.955
11600 measured reflections
7423 independent reflections
2683 reflections with *I* > 2σ(*I*)
*R*_int_ = 0.057

#### Refinement

*R*[*F* ^2^ > 2σ(*F* ^2^)] = 0.075
*wR*(*F* ^2^) = 0.193
*S* = 0.91
7423 reflections
445 parameters
2 restraints
H atoms treated by a mixture of independent and constrained refinement
Δρ_max_ = 0.29 e Å^−3^
Δρ_min_ = −0.21 e Å^−3^

### 

Data collection: *APEX2* (Bruker, 2005 ▶); cell refinement: *SAINT* (Bruker, 2005 ▶); data reduction: *SAINT*; program(s) used to solve structure: *SHELXTL* (Sheldrick, 2008 ▶); program(s) used to refine structure: *SHELXTL*; molecular graphics: *SHELXTL*; software used to prepare material for publication: *SHELXTL*.

## Supplementary Material

Crystal structure: contains datablocks global, I. DOI: 10.1107/S1600536811001218/om2397sup1.cif

Structure factors: contains datablocks I. DOI: 10.1107/S1600536811001218/om2397Isup2.hkl

Additional supplementary materials:  crystallographic information; 3D view; checkCIF report

## Figures and Tables

**Table 1:** Hydrogen-bond geometry (Å, °)

*D*—H⋯*A*	*D*—H	H⋯*A*	*D*⋯*A*	*D*—H⋯*A*
O1—H1⋯N1	0.82	1.88	2.602 (4)	146
O3—H3⋯N4	0.82	1.91	2.625 (4)	145
N2—H2⋯O4^i^	0.90 (6)	2.22 (6)	3.109 (4)	168 (7)
N5—H5⋯O2	0.90 (5)	2.00 (4)	2.899 (4)	176 (7)

Symmetry code: (i) 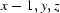.

**Fig. 1.**
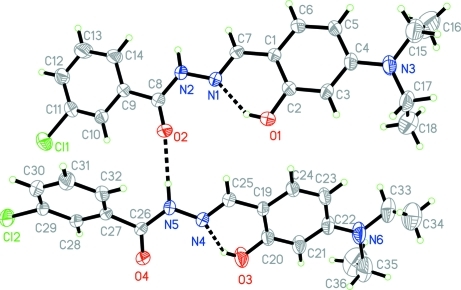
The molecular structure of the title compound with 30% probability displacement ellipsoids for non-hydrogen atoms. Hydrogen bonds are drawn as dashed lines.

**Fig. 2.**
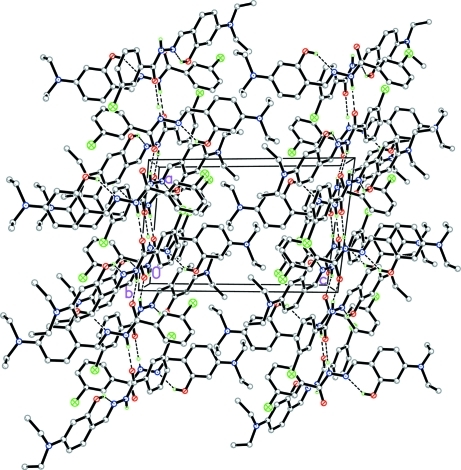
The molecular packing of the title compound. Hydrogen bonds are drawn as dashed lines.

## supplementary crystallographic information

### Comment

In the last few years, a number of Schiff bases derived from the reaction of aldehydes with benzohydrazides were prepared and structurally characterized (Bessy *et al.*, 2006; Podyachev *et al.*, 2007; Raj & Kurup, 2007; Pouralimardan *et al.*, 2007; Bacchi *et al.*, 2006; Dinda *et al.*, 2002). As a continuation of the work, in the present paper, the title new Schiff base compound, Fig. 1, is reported.

The asymmetric unit of the compound contains two independent Schiff base molecules. In each Schiff base molecule, there is an O—H···N hydrogen bond, which contributes to the planarity of the molecule. The dihedral angles between the two benzene rings in both molecules are 12.8 (3) and 27.2 (3)°, respectively. All the bond lengths are within normal values (Allen *et al.*, 1987). The molecules are linked through intermolecular N—H···O hydrogen bonds (Table 1) to form chains along the *a* axis (Fig. 2).

### Experimental

The compound was prepared and crystallized according to the literature method (Zhu, 2010). 4-Diethylamino-2-hydroxybenzaldehyde (0.193 g, 1 mmol) and 3-chlorobenzohydrazide (0.171 g, 1 mmol) were dissolved in 30 ml absolute methanol. The mixture was stirred at reflux for 10 min, and cooled to room temperature. The clear colorless solution was left to slowly evaporate in air for a few days, yielding colorless block-shaped crystals, which were collected by filtration and washed with methanol.

### Refinement

The amino H atoms were located from a difference Fourier map and refined isotropically, with the N—H distances restrained to 0.90 (1) Å. The other H atoms were positioned geometrically and refined using the riding-model approximation, with C—H = 0.93–0.97 Å, and O—H = 0.82 Å, and *U*_iso_(H) = 1.2*U*_eq_(C) or *U*_iso_(H) = 1.5*U*_eq_(methyl C and O).

### Crystal data

**Table:**

C_18_H_20_ClN_3_O_2_	*Z* = 4
*M**_r_* = 345.82	*F*(000) = 728
Triclinic, *P*1	*D*_x_ = 1.295 Mg m^−^^3^
Hall symbol: -P 1	Mo *K*α radiation, λ = 0.71073 Å
*a* = 10.087 (4) Å	Cell parameters from 1270 reflections
*b* = 12.939 (5) Å	θ = 2.3–24.5°
*c* = 14.780 (5) Å	µ = 0.23 mm^−^^1^
α = 78.408 (4)°	*T* = 298 K
β = 80.726 (4)°	Block, colorless
γ = 70.632 (4)°	0.22 × 0.20 × 0.20 mm
*V* = 1773.3 (11) Å^3^	

### Data collection

**Table:**

Bruker APEXII CCD area-detector diffractometer	7423 independent reflections
Radiation source: fine-focus sealed tube	2683 reflections with *I* > 2σ(*I*)
graphite	*R*_int_ = 0.057
ω scans	θ_max_ = 27.0°, θ_min_ = 2.2°
Absorption correction: multi-scan (*SADABS*; Bruker, 2005)	*h* = −12→12
*T*_min_ = 0.951, *T*_max_ = 0.955	*k* = −11→16
11600 measured reflections	*l* = −16→18

### Refinement

**Table:**

Refinement on *F*^2^	Primary atom site location: structure-invariant direct methods
Least-squares matrix: full	Secondary atom site location: difference Fourier map
*R*[*F*^2^ > 2σ(*F*^2^)] = 0.075	Hydrogen site location: inferred from neighbouring sites
*wR*(*F*^2^) = 0.193	H atoms treated by a mixture of independent and constrained refinement
*S* = 0.91	*w* = 1/[σ^2^(*F*_o_^2^) + (0.0717*P*)^2^] where *P* = (*F*_o_^2^ + 2*F*_c_^2^)/3
7423 reflections	(Δ/σ)_max_ < 0.001
445 parameters	Δρ_max_ = 0.29 e Å^−^^3^
2 restraints	Δρ_min_ = −0.21 e Å^−^^3^

### Special details

**Table:**

Geometry. All e.s.d.'s (except the e.s.d. in the dihedral angle between two l.s. planes) are estimated using the full covariance matrix. The cell e.s.d.'s are taken into account individually in the estimation of e.s.d.'s in distances, angles and torsion angles; correlations between e.s.d.'s in cell parameters are only used when they are defined by crystal symmetry. An approximate (isotropic) treatment of cell e.s.d.'s is used for estimating e.s.d.'s involving l.s. planes.
Refinement. Refinement of *F*^2^ against ALL reflections. The weighted *R*-factor *wR* and goodness of fit *S* are based on *F*^2^, conventional *R*-factors *R* are based on *F*, with *F* set to zero for negative *F*^2^. The threshold expression of *F*^2^ > σ(*F*^2^) is used only for calculating *R*-factors(gt) *etc*. and is not relevant to the choice of reflections for refinement. *R*-factors based on *F*^2^ are statistically about twice as large as those based on *F*, and *R*- factors based on ALL data will be even larger.

### Fractional atomic coordinates and isotropic or equivalent isotropic displacement parameters (Å^2^)

**Table:**

	*x*	*y*	*z*	*U*_iso_*/*U*_eq_	
Cl1	0.62387 (13)	−0.14819 (9)	1.18480 (9)	0.0869 (5)	
Cl2	0.89678 (13)	−0.12147 (10)	1.28108 (9)	0.0957 (5)	
N1	0.1455 (3)	0.3794 (3)	0.9448 (2)	0.0572 (9)	
N2	0.1671 (3)	0.2739 (3)	0.9968 (2)	0.0575 (9)	
N3	−0.1066 (5)	0.8362 (3)	0.6765 (3)	0.0984 (15)	
N4	0.7011 (3)	0.3310 (3)	0.8824 (2)	0.0587 (9)	
N5	0.6729 (3)	0.2805 (3)	0.9726 (2)	0.0592 (9)	
N6	0.6481 (5)	0.6139 (3)	0.4730 (3)	0.1039 (15)	
O1	0.2103 (3)	0.5586 (2)	0.8699 (2)	0.0746 (9)	
H1	0.2222	0.4963	0.9003	0.112*	
O2	0.3907 (3)	0.2608 (2)	1.01641 (19)	0.0677 (8)	
O3	0.8587 (3)	0.3508 (3)	0.7241 (2)	0.0956 (11)	
H3	0.8430	0.3298	0.7798	0.143*	
O4	0.9075 (3)	0.1993 (2)	0.98704 (19)	0.0733 (9)	
C1	−0.0103 (4)	0.5309 (3)	0.8558 (3)	0.0551 (11)	
C2	0.0842 (4)	0.5933 (3)	0.8338 (3)	0.0588 (11)	
C3	0.0517 (5)	0.6930 (3)	0.7750 (3)	0.0712 (13)	
H3A	0.1164	0.7324	0.7610	0.085*	
C4	−0.0760 (5)	0.7363 (3)	0.7358 (3)	0.0714 (13)	
C5	−0.1713 (5)	0.6755 (4)	0.7610 (3)	0.0754 (14)	
H5A	−0.2587	0.7029	0.7376	0.090*	
C6	−0.1383 (4)	0.5774 (3)	0.8191 (3)	0.0684 (13)	
H6	−0.2049	0.5396	0.8349	0.082*	
C7	0.0252 (4)	0.4233 (3)	0.9121 (3)	0.0582 (11)	
H7	−0.0404	0.3846	0.9253	0.070*	
C8	0.2939 (5)	0.2209 (3)	1.0290 (3)	0.0543 (11)	
C9	0.3083 (4)	0.1059 (3)	1.0819 (3)	0.0524 (11)	
C10	0.4398 (4)	0.0413 (3)	1.1071 (2)	0.0535 (11)	
H10	0.5157	0.0694	1.0926	0.064*	
C11	0.4587 (4)	−0.0652 (3)	1.1538 (3)	0.0563 (11)	
C12	0.3502 (5)	−0.1082 (4)	1.1782 (3)	0.0751 (14)	
H12	0.3639	−0.1792	1.2118	0.090*	
C13	0.2205 (5)	−0.0448 (4)	1.1524 (4)	0.110 (2)	
H13	0.1457	−0.0741	1.1666	0.132*	
C14	0.1978 (5)	0.0628 (4)	1.1053 (4)	0.0885 (16)	
H14	0.1080	0.1056	1.0895	0.106*	
C15	−0.2541 (6)	0.8936 (4)	0.6502 (4)	0.1111 (19)	
H15A	−0.3211	0.8725	0.6993	0.133*	
H15B	−0.2739	0.9733	0.6430	0.133*	
C16	−0.2698 (8)	0.8644 (6)	0.5664 (4)	0.160 (3)	
H16A	−0.1932	0.8731	0.5209	0.240*	
H16B	−0.3577	0.9118	0.5443	0.240*	
H16C	−0.2692	0.7886	0.5771	0.240*	
C17	−0.0019 (7)	0.8907 (5)	0.6400 (5)	0.129 (3)	
H17A	0.0494	0.8899	0.6904	0.155*	
H17B	−0.0495	0.9676	0.6163	0.155*	
C18	0.1017 (8)	0.8402 (6)	0.5645 (5)	0.171 (4)	
H18A	0.1509	0.7644	0.5874	0.256*	
H18B	0.1683	0.8807	0.5444	0.256*	
H18C	0.0525	0.8431	0.5131	0.256*	
C19	0.6100 (4)	0.4504 (3)	0.7475 (3)	0.0512 (10)	
C20	0.7394 (5)	0.4277 (3)	0.6918 (3)	0.0661 (13)	
C21	0.7527 (5)	0.4808 (4)	0.6025 (3)	0.0784 (15)	
H21	0.8405	0.4642	0.5678	0.094*	
C22	0.6361 (5)	0.5596 (4)	0.5625 (3)	0.0722 (13)	
C23	0.5055 (5)	0.5822 (3)	0.6168 (3)	0.0681 (13)	
H23	0.4256	0.6336	0.5922	0.082*	
C24	0.4960 (4)	0.5285 (3)	0.7059 (3)	0.0606 (11)	
H24	0.4083	0.5451	0.7407	0.073*	
C25	0.5939 (4)	0.3970 (3)	0.8421 (3)	0.0548 (11)	
H25	0.5043	0.4107	0.8745	0.066*	
C26	0.7832 (5)	0.2151 (3)	1.0193 (3)	0.0576 (11)	
C27	0.7429 (4)	0.1648 (3)	1.1153 (3)	0.0531 (11)	
C28	0.8251 (4)	0.0584 (3)	1.1485 (3)	0.0595 (12)	
H28	0.9004	0.0196	1.1107	0.071*	
C29	0.7937 (5)	0.0109 (4)	1.2382 (3)	0.0661 (13)	
C30	0.6833 (5)	0.0675 (4)	1.2948 (3)	0.0780 (14)	
H30	0.6631	0.0345	1.3551	0.094*	
C31	0.6028 (5)	0.1733 (4)	1.2619 (3)	0.0724 (13)	
H31	0.5284	0.2120	1.3004	0.087*	
C32	0.6316 (5)	0.2225 (3)	1.1718 (3)	0.0621 (12)	
H32	0.5765	0.2938	1.1496	0.074*	
C33	0.5259 (6)	0.6822 (4)	0.4238 (3)	0.108 (2)	
H33A	0.5483	0.6773	0.3581	0.130*	
H33B	0.4471	0.6537	0.4466	0.130*	
C34	0.4860 (7)	0.7949 (5)	0.4356 (4)	0.134 (2)	
H34A	0.4598	0.8005	0.5003	0.201*	
H34B	0.4071	0.8373	0.4012	0.201*	
H34C	0.5639	0.8234	0.4134	0.201*	
C35	0.8030 (8)	0.6106 (5)	0.4205 (4)	0.124 (2)	
H35A	0.7998	0.6788	0.3778	0.149*	
H35B	0.8706	0.5981	0.4644	0.149*	
C36	0.8349 (10)	0.5222 (7)	0.3741 (5)	0.195 (4)	
H36A	0.8597	0.4543	0.4172	0.292*	
H36B	0.9131	0.5236	0.3277	0.292*	
H36C	0.7544	0.5269	0.3448	0.292*	
H2	0.092 (5)	0.249 (6)	1.003 (5)	0.234*	
H5	0.584 (3)	0.277 (6)	0.988 (5)	0.234*	

### Atomic displacement parameters (Å^2^)

**Table:**

	*U*^11^	*U*^22^	*U*^33^	*U*^12^	*U*^13^	*U*^23^
Cl1	0.0701 (8)	0.0716 (8)	0.1025 (10)	−0.0038 (6)	−0.0293 (7)	0.0090 (7)
Cl2	0.0751 (9)	0.0812 (9)	0.1098 (10)	−0.0245 (7)	−0.0273 (7)	0.0472 (7)
N1	0.051 (2)	0.055 (2)	0.061 (2)	−0.0210 (17)	−0.0106 (17)	0.0139 (17)
N2	0.049 (2)	0.052 (2)	0.063 (2)	−0.0155 (17)	−0.0095 (18)	0.0138 (17)
N3	0.092 (3)	0.074 (3)	0.128 (4)	−0.039 (3)	−0.058 (3)	0.046 (3)
N4	0.055 (2)	0.059 (2)	0.058 (2)	−0.0243 (18)	−0.0094 (18)	0.0131 (18)
N5	0.057 (2)	0.063 (2)	0.053 (2)	−0.0246 (19)	−0.0149 (18)	0.0184 (18)
N6	0.098 (4)	0.102 (3)	0.066 (3)	−0.002 (3)	0.006 (3)	0.031 (2)
O1	0.0512 (18)	0.083 (2)	0.087 (2)	−0.0310 (17)	−0.0220 (16)	0.0219 (17)
O2	0.0512 (18)	0.0688 (19)	0.083 (2)	−0.0303 (16)	−0.0139 (15)	0.0136 (16)
O3	0.064 (2)	0.097 (2)	0.077 (2)	0.0121 (18)	0.0031 (17)	0.024 (2)
O4	0.0496 (19)	0.085 (2)	0.076 (2)	−0.0279 (17)	−0.0101 (16)	0.0232 (16)
C1	0.046 (2)	0.051 (2)	0.066 (3)	−0.019 (2)	−0.013 (2)	0.010 (2)
C2	0.049 (3)	0.060 (3)	0.065 (3)	−0.020 (2)	−0.018 (2)	0.009 (2)
C3	0.069 (3)	0.060 (3)	0.087 (3)	−0.037 (2)	−0.027 (3)	0.026 (2)
C4	0.072 (3)	0.055 (3)	0.084 (3)	−0.025 (3)	−0.028 (3)	0.021 (2)
C5	0.059 (3)	0.070 (3)	0.096 (4)	−0.029 (3)	−0.034 (3)	0.027 (3)
C6	0.053 (3)	0.067 (3)	0.086 (3)	−0.028 (2)	−0.021 (2)	0.016 (2)
C7	0.047 (3)	0.055 (3)	0.072 (3)	−0.024 (2)	−0.008 (2)	0.008 (2)
C8	0.049 (3)	0.054 (3)	0.055 (3)	−0.016 (2)	−0.005 (2)	0.002 (2)
C9	0.045 (2)	0.054 (3)	0.054 (3)	−0.014 (2)	−0.010 (2)	0.002 (2)
C10	0.055 (3)	0.054 (3)	0.053 (2)	−0.020 (2)	−0.011 (2)	−0.003 (2)
C11	0.060 (3)	0.050 (3)	0.057 (3)	−0.009 (2)	−0.018 (2)	−0.008 (2)
C12	0.074 (3)	0.053 (3)	0.096 (4)	−0.022 (3)	−0.026 (3)	0.012 (2)
C13	0.076 (4)	0.075 (4)	0.177 (6)	−0.042 (3)	−0.041 (4)	0.041 (4)
C14	0.055 (3)	0.067 (3)	0.135 (4)	−0.028 (3)	−0.030 (3)	0.034 (3)
C15	0.128 (5)	0.089 (4)	0.117 (5)	−0.042 (4)	−0.036 (4)	0.014 (4)
C16	0.228 (8)	0.231 (8)	0.073 (4)	−0.150 (7)	−0.048 (5)	0.019 (5)
C17	0.133 (6)	0.091 (4)	0.178 (7)	−0.076 (5)	−0.086 (5)	0.072 (5)
C18	0.136 (7)	0.190 (8)	0.161 (7)	−0.075 (6)	−0.026 (6)	0.079 (6)
C19	0.051 (3)	0.045 (2)	0.049 (2)	−0.011 (2)	−0.008 (2)	0.0059 (19)
C20	0.058 (3)	0.060 (3)	0.060 (3)	−0.004 (2)	−0.003 (2)	0.007 (2)
C21	0.066 (3)	0.080 (3)	0.057 (3)	0.001 (3)	0.010 (2)	0.009 (3)
C22	0.084 (4)	0.064 (3)	0.054 (3)	−0.013 (3)	−0.007 (3)	0.007 (2)
C23	0.067 (3)	0.061 (3)	0.058 (3)	−0.004 (2)	−0.015 (2)	0.011 (2)
C24	0.055 (3)	0.058 (3)	0.058 (3)	−0.009 (2)	−0.007 (2)	0.002 (2)
C25	0.054 (3)	0.054 (2)	0.054 (3)	−0.019 (2)	−0.008 (2)	0.002 (2)
C26	0.051 (3)	0.054 (3)	0.067 (3)	−0.026 (2)	−0.014 (2)	0.017 (2)
C27	0.047 (3)	0.054 (3)	0.060 (3)	−0.026 (2)	−0.015 (2)	0.012 (2)
C28	0.044 (2)	0.063 (3)	0.067 (3)	−0.022 (2)	−0.012 (2)	0.014 (2)
C29	0.050 (3)	0.064 (3)	0.076 (3)	−0.024 (2)	−0.022 (2)	0.030 (3)
C30	0.074 (4)	0.090 (4)	0.070 (3)	−0.040 (3)	−0.018 (3)	0.023 (3)
C31	0.072 (3)	0.080 (3)	0.060 (3)	−0.021 (3)	−0.004 (3)	−0.006 (3)
C32	0.061 (3)	0.058 (3)	0.066 (3)	−0.022 (2)	−0.019 (2)	0.008 (2)
C33	0.149 (6)	0.082 (4)	0.069 (4)	−0.018 (4)	−0.005 (4)	0.012 (3)
C34	0.179 (7)	0.096 (5)	0.110 (5)	−0.024 (5)	−0.008 (4)	−0.019 (4)
C35	0.191 (8)	0.102 (5)	0.078 (4)	−0.049 (5)	−0.032 (5)	0.011 (4)
C36	0.303 (12)	0.218 (9)	0.105 (6)	−0.123 (9)	−0.037 (6)	−0.035 (6)

### Geometric parameters (Å, °)

**Table:**

Cl1—C11	1.732 (4)	C15—H15A	0.9700
Cl2—C29	1.736 (4)	C15—H15B	0.9700
N1—C7	1.285 (4)	C16—H16A	0.9600
N1—N2	1.392 (4)	C16—H16B	0.9600
N2—C8	1.347 (5)	C16—H16C	0.9600
N2—H2	0.90 (6)	C17—C18	1.493 (9)
N3—C4	1.380 (5)	C17—H17A	0.9700
N3—C17	1.435 (6)	C17—H17B	0.9700
N3—C15	1.500 (6)	C18—H18A	0.9600
N4—C25	1.283 (4)	C18—H18B	0.9600
N4—N5	1.392 (4)	C18—H18C	0.9600
N5—C26	1.349 (5)	C19—C24	1.389 (5)
N5—H5	0.90 (5)	C19—C20	1.401 (5)
N6—C22	1.375 (5)	C19—C25	1.438 (5)
N6—C33	1.462 (6)	C20—C21	1.369 (5)
N6—C35	1.620 (7)	C21—C22	1.399 (5)
O1—C2	1.360 (4)	C21—H21	0.9300
O1—H1	0.8200	C22—C23	1.401 (6)
O2—C8	1.222 (4)	C23—C24	1.365 (5)
O3—C20	1.366 (4)	C23—H23	0.9300
O3—H3	0.8200	C24—H24	0.9300
O4—C26	1.232 (4)	C25—H25	0.9300
C1—C6	1.379 (5)	C26—C27	1.490 (5)
C1—C2	1.404 (5)	C27—C32	1.382 (5)
C1—C7	1.435 (5)	C27—C28	1.387 (5)
C2—C3	1.374 (5)	C28—C29	1.380 (5)
C3—C4	1.393 (5)	C28—H28	0.9300
C3—H3A	0.9300	C29—C30	1.371 (6)
C4—C5	1.395 (5)	C30—C31	1.376 (6)
C5—C6	1.354 (5)	C30—H30	0.9300
C5—H5A	0.9300	C31—C32	1.385 (5)
C6—H6	0.9300	C31—H31	0.9300
C7—H7	0.9300	C32—H32	0.9300
C8—C9	1.507 (5)	C33—C34	1.418 (6)
C9—C14	1.372 (5)	C33—H33A	0.9700
C9—C10	1.377 (5)	C33—H33B	0.9700
C10—C11	1.377 (5)	C34—H34A	0.9600
C10—H10	0.9300	C34—H34B	0.9600
C11—C12	1.354 (5)	C34—H34C	0.9600
C12—C13	1.361 (6)	C35—C36	1.372 (8)
C12—H12	0.9300	C35—H35A	0.9700
C13—C14	1.388 (5)	C35—H35B	0.9700
C13—H13	0.9300	C36—H36A	0.9600
C14—H14	0.9300	C36—H36B	0.9600
C15—C16	1.411 (7)	C36—H36C	0.9600
			
C7—N1—N2	115.4 (3)	C18—C17—H17B	108.6
C8—N2—N1	118.4 (3)	H17A—C17—H17B	107.6
C8—N2—H2	128 (5)	C17—C18—H18A	109.5
N1—N2—H2	114 (5)	C17—C18—H18B	109.5
C4—N3—C17	122.1 (4)	H18A—C18—H18B	109.5
C4—N3—C15	120.2 (4)	C17—C18—H18C	109.5
C17—N3—C15	117.6 (4)	H18A—C18—H18C	109.5
C25—N4—N5	116.2 (3)	H18B—C18—H18C	109.5
C26—N5—N4	118.1 (3)	C24—C19—C20	115.9 (4)
C26—N5—H5	123 (5)	C24—C19—C25	121.2 (4)
N4—N5—H5	116 (5)	C20—C19—C25	122.9 (4)
C22—N6—C33	123.0 (5)	O3—C20—C21	116.7 (4)
C22—N6—C35	119.7 (4)	O3—C20—C19	121.5 (4)
C33—N6—C35	117.2 (4)	C21—C20—C19	121.9 (4)
C2—O1—H1	109.5	C20—C21—C22	121.0 (4)
C20—O3—H3	109.5	C20—C21—H21	119.5
C6—C1—C2	116.6 (3)	C22—C21—H21	119.5
C6—C1—C7	121.5 (4)	N6—C22—C21	121.6 (5)
C2—C1—C7	121.8 (3)	N6—C22—C23	120.5 (4)
O1—C2—C3	117.3 (4)	C21—C22—C23	117.9 (4)
O1—C2—C1	121.8 (3)	C24—C23—C22	119.7 (4)
C3—C2—C1	120.9 (4)	C24—C23—H23	120.2
C2—C3—C4	121.5 (4)	C22—C23—H23	120.2
C2—C3—H3A	119.3	C23—C24—C19	123.6 (4)
C4—C3—H3A	119.3	C23—C24—H24	118.2
N3—C4—C3	120.7 (4)	C19—C24—H24	118.2
N3—C4—C5	122.2 (4)	N4—C25—C19	121.0 (4)
C3—C4—C5	117.1 (4)	N4—C25—H25	119.5
C6—C5—C4	121.0 (4)	C19—C25—H25	119.5
C6—C5—H5A	119.5	O4—C26—N5	123.6 (4)
C4—C5—H5A	119.5	O4—C26—C27	122.1 (4)
C5—C6—C1	122.8 (4)	N5—C26—C27	114.4 (4)
C5—C6—H6	118.6	C32—C27—C28	120.2 (4)
C1—C6—H6	118.6	C32—C27—C26	122.0 (4)
N1—C7—C1	121.4 (4)	C28—C27—C26	117.8 (4)
N1—C7—H7	119.3	C29—C28—C27	119.2 (4)
C1—C7—H7	119.3	C29—C28—H28	120.4
O2—C8—N2	123.8 (4)	C27—C28—H28	120.4
O2—C8—C9	121.9 (4)	C30—C29—C28	121.0 (4)
N2—C8—C9	114.3 (4)	C30—C29—Cl2	119.3 (4)
C14—C9—C10	118.9 (4)	C28—C29—Cl2	119.7 (4)
C14—C9—C8	123.4 (4)	C29—C30—C31	119.7 (4)
C10—C9—C8	117.8 (4)	C29—C30—H30	120.2
C11—C10—C9	119.8 (4)	C31—C30—H30	120.2
C11—C10—H10	120.1	C30—C31—C32	120.5 (4)
C9—C10—H10	120.1	C30—C31—H31	119.8
C12—C11—C10	121.8 (4)	C32—C31—H31	119.8
C12—C11—Cl1	117.7 (3)	C27—C32—C31	119.5 (4)
C10—C11—Cl1	120.4 (4)	C27—C32—H32	120.3
C11—C12—C13	118.3 (4)	C31—C32—H32	120.3
C11—C12—H12	120.8	C34—C33—N6	111.9 (5)
C13—C12—H12	120.8	C34—C33—H33A	109.2
C12—C13—C14	121.3 (4)	N6—C33—H33A	109.2
C12—C13—H13	119.4	C34—C33—H33B	109.2
C14—C13—H13	119.4	N6—C33—H33B	109.2
C9—C14—C13	119.8 (4)	H33A—C33—H33B	107.9
C9—C14—H14	120.1	C33—C34—H34A	109.5
C13—C14—H14	120.1	C33—C34—H34B	109.5
C16—C15—N3	110.3 (6)	H34A—C34—H34B	109.5
C16—C15—H15A	109.6	C33—C34—H34C	109.5
N3—C15—H15A	109.6	H34A—C34—H34C	109.5
C16—C15—H15B	109.6	H34B—C34—H34C	109.5
N3—C15—H15B	109.6	C36—C35—N6	101.9 (6)
H15A—C15—H15B	108.1	C36—C35—H35A	111.4
C15—C16—H16A	109.5	N6—C35—H35A	111.4
C15—C16—H16B	109.5	C36—C35—H35B	111.4
H16A—C16—H16B	109.5	N6—C35—H35B	111.4
C15—C16—H16C	109.5	H35A—C35—H35B	109.2
H16A—C16—H16C	109.5	C35—C36—H36A	109.5
H16B—C16—H16C	109.5	C35—C36—H36B	109.5
N3—C17—C18	114.6 (6)	H36A—C36—H36B	109.5
N3—C17—H17A	108.6	C35—C36—H36C	109.5
C18—C17—H17A	108.6	H36A—C36—H36C	109.5
N3—C17—H17B	108.6	H36B—C36—H36C	109.5

### Hydrogen-bond geometry (Å, °)

**Table:**

*D*—H···*A*	*D*—H	H···*A*	*D*···*A*	*D*—H···*A*
O1—H1···N1	0.82	1.88	2.602 (4)	146
O3—H3···N4	0.82	1.91	2.625 (4)	145
N2—H2···O4^i^	0.90 (6)	2.22 (6)	3.109 (4)	168 (7)
N5—H5···O2	0.90 (5)	2.00 (4)	2.899 (4)	176 (7)

Symmetry codes: (i) *x*−1, *y*, *z*.
